# Supplementary material for: Study protocol: The effect of a Medication Coordinator on the quality of patients’ medication treatment (MEDCOOR)—Randomized controlled trial
Source: PLoS One. 2024 Nov 26;19(11):e0314023. doi: 10.1371/journal.pone.0314023 (PMC11593754; doi:10.1371/journal.pone.0314023)
Supplement: S2 File — The do file used for the power calculation. (PDF) [file pone.0314023.s002.pdf]

S2 Appendix - The Do File from STATA/BE used to the power calculation

The commandoes to be used:

```
clear

local mc=500

set obs `mc'


generate Registrering=.

generate powerreg=.

generate Powerreg=.

quietly{

forvalues i=1(1)`mc'{

if
`i'==10*`mc'/100|`i'==20*`mc'/100|`i'==30*`mc'/100|`i'==40*`mc'/100|`i'==50*`mc'/100|`i'==60*`mc'/100
|`i'==70*`mc'/100|`i'==80*`mc'/100|`i'==90*`mc'/100{

noisily display `i'/`mc'*100 "% done"

}

preserve

clear

set seed `i'

set obs 140 //Antallet af patienter

gen id=_n

gen group=rbinomial(1,0.5)

gen age=rnormal(70,10)

gen logitp=1.1*age-70

gen p=exp(logitp)/(exp(logitp)+1)

gen dropout=rbinomial(1,p)

expand 2

bysort id: gen time=_n

gen mean=.
```

```
gen variance=.
replace mean= 0.15 if time==1 & group==0
replace mean= 0.10 if time==2 & group==0
replace mean= 0.12 if time==1 & group==1
replace mean= 0.13 if time==2 & group==1

replace variance= 0.05^2 if time==1 & group==0
replace variance= 0.05^2 if time==2 & group==0
replace variance= 0.05^2 if time==1 & group==1
replace variance= 0.05^2 if time==2 & group==1
gen a=(1-mean)/variance-1/mean
replace a=a*mean^2
gen b=1/mean-1
replace b=b*a
gen outcome=rbeta(a,b)
summarize outcome if time==1 & group==0
summarize outcome if time==2 & group==0
summarize outcome if time==1 & group==1
summarize outcome if time==2 & group==1
replace outcome=. if dropout==1
betareg outcome i.group#i.time
estimates store m1
betareg outcome i.time
estimates store m0
lrtest m1 m0
matrix define Areg=r(p)
restore
replace Registrering=Areg[1,1] in `i'
replace powerreg=1 if Registrering<0.05 //Significance level
replace Powerreg=sum(powerreg) //Convergence
```

```
}

replace powerreg=0 if powerreg==.
generate iteration=_n
replace Powerreg=Powerreg/iteration
}

tab powerreg
twoway (connected Powerreg iteration)
```
