# Supplementary material for: Study protocol: The effect of a Medication Coordinator on the quality of patients’ medication treatment (MEDCOOR)—Randomized controlled trial
Source: PLoS One. 2024 Nov 26;19(11):e0314023. doi: 10.1371/journal.pone.0314023 (PMC11593754; doi:10.1371/journal.pone.0314023)
Supplement: S3 File — (PDF) [file pone.0314023.s003.pdf]

Effekten af en medicinkoordinator på kvaliteten af lægemiddelbehandlingen: Et randomiseret, kontrolleret studie

Ph.d.-studerende:

Maja Schlünsen, Cand. Pharm.

Mail: maja.schlunsen@rsyd.dk

Hovedvejleder:

Lene Juel Kjeldsen, Lektor, Forskningsleder, Ph.d.

Hospital Pharmacy Research Unit, Institut for Regional Sundhedsforskning,

Syddansk Universitet

Sygehusapoteker, Sygehus Sønderjylland

Medvejleder:

Trine Graabæk

Postdoc, Ph.d.

Sygehusapotek Fyn, Odense Universitetshospital

Institut for Sundhedstjenesteforskning, Syddansk Universitet

Formål

Formålet med projektet er at undersøge både feasibility samt effekt af en medicinkoordinator for indlagte pa-tienter målt ved antallet af potentielle uhensigtsmæssige lægemidler ved udskrivelse og 6 måneder efter. Me-dicinkoordinatoren tager udgangspunkt i patientens individuelle lægemiddelbehandling samt -behov med fo-kus på overgangen fra hospital til eget hjem. Medicinkoordinatorens rolle er at fungere som patientens og/eller pårørendes samarbejdspartner i forbindelse med optimering og formidling af lægemiddelbehandling. Til for-målet udvikles hæftet *Min medicinplan*.

Forskningsplan

Ph.d.-projektet består af tre studier: 1. Udvikling af hæftet *Min medicinplan*, 2. Evaluering af interventionens feasibility og 3. Randomiseret, kontrolleret studie (RCT) af medicingennemgang med medicinkoordinator.

<u>Studie 1- Udvikling af hæftet Min medicinplan</u>

I projektet ønskes der at udvikle hæftet *Min medicinplan* med plads til tanker, lægemidler, doseringer, symptomer, fremtidige aftaler, hvem er behandlingsansvarlig læge, hvem skal patienten kontakte, etc.(1,2).

Hahn-Goldberg et al har udviklet et udskrivelsesværktøj, hvori patienter og pårørende kan notere ændringer, seponering af lægemidler samt fremtidige aftaler i forhold til konsultationer, og hvilken rolle apoteket kan have(3). Dette værktøj er dog ikke tilpasset en dansk kontekst. I Danmark blev der i 2006 udviklet *Patientens bog – en guide til et sikkert patientforløb*, som er en bog på 131 sider med en bred vifte af gode råd og plads til notater fra patienten og pårørende(4). Der ønskes dog i dette studie at udvikle et mindre hæfte, som kunne være et overskueligt værktøj for patienten. Til udviklingen udføres semi-strukturerede uformelle patientinter-views(5) vedrørende patienternes udskrivelsesproces for at klarlægge patienternes forventninger og ønsker til information omkring lægemidler. Der forventes at inkludere ti patienter afhængig af datamætning. Ydermere observeres lægernes og sygeplejerskernes rolle i forbindelse med udskrivelsesprocessen.

Til evaluering af *Min medicinplan* udføres uformelle interviewes med patienter fra samme afdeling som de indledede observationer og uformelle interview fokus på om *Min medicinplan* kan være behjælpelig i sektorovergangen for at opnå et relevant, anvendeligt og praksisnært værktøj.

<u>Studie 2 – Evaluering af interventionens feasibility</u>

Studie 2 har til formål at evaluere modellen for medicinkoordinatorens intervention i forbindelse med medicin-gennemgange. Det er den ph.d.-studerende som i studiet er medicinkoordinatoren. Medicinkoordinatorens rolle er følgende: At øge patienternes empowerment i forhold til brugen af lægemidler og derved øge compliance, at fungere som en samarbejdspartner for patienten ved gensidig tillid mellem patient og medicinkoordinator, at gennemføre og kommunikere resultatet af medicingennemgangen til hospitalslægen, som tager stilling til ændringerne, og efterfølgende at formidle eventuelle ændringer samt bevæggrunden herfor til patienten. I forlængelse heraf skal medicinkoordinatoren formidle epikrisen til patienten, således at patienten ved, hvilke informationer der bliver givet videre til alment praktiserende læge(6–8). I processen anvendes *Min medicin-plan*, der udvikles i studie 1. Det forventes dermed, at overgangen fra hospitalet til hjemmet gøres lettere, da patienten har forventningsafstemt i forhold til, hvilken læge der er ansvarlig for behandlingsforløbet. Ydermere skal medicinkoordinatoren i udvalgte situationer fungere som et bindeled mellem patient og alment praktiserende læge/speciallæge og derved øge kommunikationen på tværs af sektorer. Evalueringen foretages ved at vurdere de procesmål, som forekom under medicingennemgangen. Dette gøres ved, at medicinkoordinatoren dokumenterer aftalte aktiviteter og implementeringen heraf. Ydermere udføres patientinterview med et udvalg af de inkluderede patienter for at opnå patienternes perspektiv på medicinkoordinatorintervention.

2.1 Målepunkter for feasibility af medicinkoordinatorinterventionen.

Procesmål:

o Hvordan leverede vi medicingennemgangen?

o Forekom der overordnede behov som gik på tværs af patienterne?

o Forekom der individuelle behov?

o Inddeling af tematikker: complians, bekymringer, inhalationsteknik etc.

Hvilke(n) typer af uhensigtsmæssige lægemidler blev reduceret med udgangspunkt i den nyeste ud-gave af Seponeringslisten(9) og *Screening tool of older people's prescriptions* (STOPP-kriterierne)(10)

o Hvilke lægemidler ønskede patienterne at snakke om under medicingennemgangen samt hvilke lægemidler ønskede patienten reelt at stoppe med.

o Hvilke lægemidler var eventuelt aldrig på tale?

Tilfredshed og implementering:

o Hvad synes patienten om bidraget fra en medicinkoordinator?

o Hvordan kan implementering af en medicinkoordinator forbedres?

Studie 3 – Effekten af medicinkoordinator i forbindelse med medicingennemgang

Formålet med dette studie er at undersøge effekten af en medicinkoordinator, hvilket udføres som et randomiseret, kontrolleret studie, hvor følgende nul-hypotese testes: Der er ikke en reduktion i antallet af listede PIMs mellem interventions- og kontrolgruppen efter medicingennemgang ved brug af den nyeste udgave af Seponeringslisten(9) samt STOPP-kriterierne(10) efter 6 måneder. Studiet vil blive gennemført i henhold til CONSORT guidelines for at sikre transparens og kvalitet i gennemførslen og resultaterne af projektet(11). Interventionen er en kompleks intervention, hvor de forskellige elementer tilsammen udgør den samlede intervention. Interventionsgruppen får en medicingennemgang i samarbejde med en medicinkoordinator, mens kontrolgruppen får standardbehandling.

Patienterne vil blive inkluderet under indlæggelse på Sygehus Sønderjylland (SHS) af den ph.d.-studerende i samarbejde med geriatrisk afdeling. Forløbet i interventionsgruppen er illustreret i figur 1.

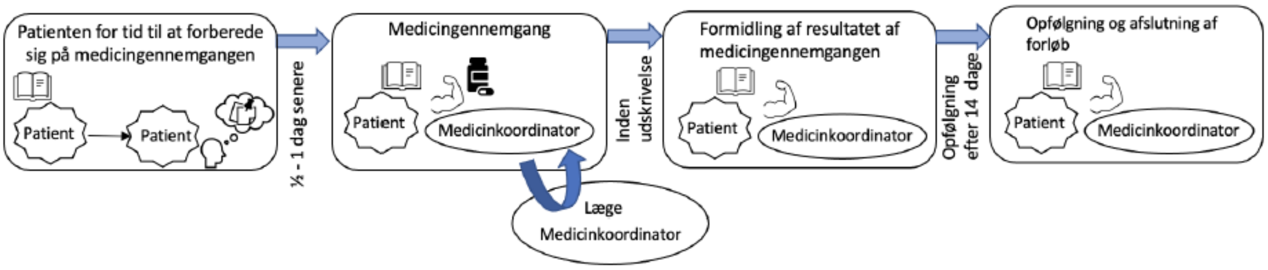

Figur 1 Forløbet i forbindelse med medicingennemgangen. Første kasse illustrerer, at patienterne får udleveret hæftet, Min medicin-plan, således de får tid til at forberede sig på medicingennemgangen. Anden kasse illustrerer udførslen af medicingennemgangen og medicinkoordinatorens drøftelse med hospitalslægen. Tredje kasse illustrerer formidlingen af resultatet af medicingennemgangen, og fjerde og sidste kasse illustrerer den afsluttende opfølgning mellem patient og medicinkoordinator. Symboler: hæftet illustrerer Min medicinplan som udleveres til patienten, Armen illustrerer patientempowerment. Medicinflasken illustrerer det fokus der er på lægemiddelbehandlingen.

Patienten bliver introduceret til medicingennemgangen, som foretages ½ - 1 dag senere. Dette giver patienten mulighed for at forberede sig, da patienten får udleveret materiale med spørgsmål, som kan give anledning til refleksion i forhold til medicingennemgangen.

Medicingennemgangen udføres ved brug af den motiverende samtale, hvor fokus er på patientens ønsker og formålet med lægemiddelbehandlingen(12). Derudover opfordres patienten til at skrive notater i hæftet *Min medicinplan*. Dette er en måde at synliggøre overfor patienten, hvad patienten selv har ytret af ønsker(12). Hospitalslægen informeres om de forandringer/ændringer som patienten fandt vigtige under medicingennemgangen og inden udskrivelse formidles resultatet af medicingennemgangen til patienten med opfordringen til at skrive medicinændringer ned i *Min medicinplan* og medbringe denne ved fremtidige konsultationer hos praktiserende læge. Medicinkoordinatoren tager kontakt til patienten omkring 14 dage efter udskrivelse for at følge op på aftaler indgået i forbindelse med medicingennemgangen.

Inklusionskriterierne er, at patienten selv varetager dispensering samt administration af lægemidler samt enten administrerer 8 lægemiddelstoffer eller 5 lægemiddelstoffer med mindst 10 daglige administrationer.

3.1 Det primære effektmål for interventionen

Det primære effektmål er at reducere antallet af PIMs 6 måneder efter udskrivelse vurderet ud fra lægemidlerne angivet på den nyeste version af Seponeringslisten(9) og STOPP-kriterierne(10). Efter 6 måneder vurderes patientens fælles medicinkort (FMK), om ændringer foretager under medicingennemgangen fortsat, er gældende, samt om seponeret behandling er genintroduceret.

De informationer som der indhentes er patientens aktuelle lægemidler på ATC-kode niveau. Yderligere indhentes informationer om patientens alder og køn.

3.1.2 Styrkeberegning

Med baggrund i litteraturen udføres styrkeberegning for at vurdere antallet af inkluderede patienter med udgangspunkt i det primære endepunkt, som er PIMs vurderet ud fra Seponeringslisten(9) samt STOPP-kriterierne(10). Til beregningen benyttes statistiskprogrammet STATA, hvor styrken sættes til 80 % samt et signifikansniveau på 5 %(13). I studiet af San-José et al, hvor interventionen var at seponere PIMs og evaluere om disse forblev seponeret efter 6 måneder(14), blev angivet en middelværdi for antallet af PIMs før interventionen er 1,4 med en spredning på 1,4 samt en middelværdi på 0,7 PIMs med en spredning på 1,1 efter 6 måneder(14). Dette betyder ifølge styrkeberegningen, at der skal inkluderes 52 patienter i hver gruppe. I studiet af Coronado-Vazquez et al udføres en intervention, hvor standardydelsen er en medicingennemgang og interventionen er en medicingennemgang ved brug af patientinvolvering(15). Interventionsgruppen startede med 1,39 PIMs og efter medicingennemgangen reduceres dette til 0,69 PIMs(15), hvorfor der ifølge resultatet af en styrkeberegning på baggrund af dette skal inkluderes 41 patienter i hver arm. Der skal inkluderes 67 patienter i hver arm, såfremt der tages udgangspunkt i kontrolgruppen, hvor udgangspunktet er 1,65 PIMs som reduceres til 1,16 PIMs. For at modregne et eventuelt frafald vurderes der, at der skal inkluderes 10 % ekstra, hvorfor der med udgangspunkt i den mest konservative styrkeberegning på 67 patienter forventes at inkludere 80 patienter i hver arm.

3.2 Det sekundære effektmål for interventionen

Ydermere undersøges patientens livskvalitet samt oplevet medicinbyrde. Til formålet anvendes spørgeskemaet Depression List(16,17). Dette spørgeskema indeholder 15 spørgsmål, som i sin oprindelse undersøger patienter for mulig depression, men spørgsmålene relaterer sig alle til andre aspekter, som har betydning for livskvaliteten(16). Der tilføjes en visuel analog skala (VAS) angående oplevet medicinbyrde(18). VAS kan benyttes til at vurdere subjektive fænomener og disse kan derved konverteres til numerisk data(19).

Etiske overvejelser

Videnskabsetisk Komité for Region Syddanmark er blevet rådført i forhold til om det beskrevne projekt skal godkendes af Videnskabsetisk Komité. Dog skal de tre studier registreres under Regionens Interne Fortegnelse, da der arbejdes med personfølsomme data(8). Forud for deltagelse i delstudie 1, delstudie 2 og delstudie 3 underskriver deltagerne informeret samtykke, hvor af det fremgår at deltagelse i studiet er frivilligt samt hvilke informationer der indsamles i hver af studierne. Det bliver tydeliggjort for patienterne at de kan trække deres samtykke tilbage på et hvert givet tidspunkt uden at give en forklaring.

Informationer og oplysninger der indhentes om patienterne:

I delstudie 1 indhentes oplysninger om patienters alder, køn og antallet af lægemidler ordineret i FMK. Yderligere, observeres patienterne i forbindelse med deres udskrivelse for at opnå viden omkring udskrivelsessamtaler samt et uformelt patientinterview efterfølgende. Disse samtaler bliver ikke lydoptaget,

da observationer udelukkende har til formål at få en forståelse af, hvordan udskrivelser forløber og hvad der bliver talt om i forhold til medicinen. Data opbevares i anonymiseret form på et sikret drev.

I delstudie 2 indhentes oplysninger om patienten alder og køn samt deres personlige perspektiver på medicin-koordinator-ydelsen. Disse interview bliver lydoptaget, da der er behov for ordrette citater. Data opbevares i anonymiseret form på et sikret drev.

I delstudie 3 indhentes oplysninger om patientens alder, køn samt aktuelle medicinliste ved indlæggelse, ved udskrivelse samt 6 måneder efter udskrivelse på ATC-kode niveau. Yderligere, besvarer patienterne et spørgeskema vedrørende livskvalitet samt oplevet medicinbyrde, hvor data bruges til at vurdere effekten af interventionen som helhed og ikke på individuelt niveau. Data opbevares i anonymiseret form på et sikret drev.

Referencer

1.Skovgaard AK, Fuglsang C, Graae EM, Haugaard IB, Christoffersen LB, Ljungmann R, m.fl. Sundhedsvæsenet ifølge danskerne [Internet]. 2016 [henvist 23. januar 2022] s. 72. Tilgængelig hos: https://www.tryghed.dk/viden/publikatio-ner/sundhed/sundhedsvaesenet-ifoelge-danskerne

2.Dansk Selskab for Patientsikkerhed, TrygFonden. Patientens bog - En guide til et sikkert patientforløb [Internet]. 2. udg. Bd. 2. Dansk Selskab for Patientsikkerhed og TrygFonden og Lindhard og Ringhof Forlag A/S; [henvist 23. januar 2022]. 131 s. Tilgængelig hos: https://patientsikkerhed.dk/content/uploads/2015/12/patientens_2.udg1opl.pdf

3.Hahn-Goldberg S, Chaput A, Rosenberg-Yunger Z, Lunsky Y, Okrainec K, Guilcher S, m.fl. Tool development to im-prove medication information transfer to patients during transitions of care: A participatory action research and de-sign thinking methodology approach. Res Soc Adm Pharm RSAP. januar 2022;18(1):2170–7.

4.Dansk Selskab for Patientsikkerhed, TrygFonden. Patientens bog - En guide til et sikkert patientforløb [Internet]. 2. udg. Bd. 2. Dansk Selskab for Patientsikkerhed og TrygFonden og Lindhard og Ringhof Forlag A/S; [henvist 23. januar 2022]. 131 s. Tilgængelig hos: https://patientsikkerhed.dk/content/uploads/2015/12/patientens_2.udg1opl.pdf

5.Brinkmann S, Tanggaard L. Kvalitative metoder: en grundbog. Kbh.: Hans Reitzel; 2015.

6.Patientovergange - Styrelsen for Patientsikkerhed [Internet]. [henvist 18. januar 2022]. Tilgængelig hos: https://stps.dk/da/laering/risikoomraader/patientovergange/

7.Sundheds- og Ældreministeriet. Vejledning om epikriser [Internet]. nov 30, 2018. Tilgængelig hos: https://www.rets-information.dk/eli/retsinfo/2018/10036

8.Sundheds- og Ældreministeriet. Bekendtgørelse af lov om videnskabsetisk behandling af sundhedsvidenskabelige forskningsprojekter og sundhedsdatavidenskabelige forskningsprojekter [Internet]. sep 1, 2020. Tilgængelig hos: https://www.retsinformation.dk/eli/lta/2020/1338

9.Seponeringslisten 2022 - anbefalinger til seponering af hyppigt anvendte lægemidler hos voksne||Indledning [Inter-net]. [henvist 18. januar 2022]. Tilgængelig hos: https://app.magicapp.org/#/guideline/5773

10.O'Mahony D, O'Sullivan D, Byrne S, O'Connor MN, Ryan C, Gallagher P. STOPP/START criteria for potentially inap-propriate prescribing in older people: version 2. Age Ageing. marts 2015;44(2):213–8.

11.Cuschieri S. The CONSORT statement. Saudi J Anaesth. april 2019;13(Suppl 1):S27–30.

12.Rosdahl G. Hvordan kan man gennem samtale motivere men- nesker til adfærdsændringer? Det er det spørgsmål, den motiverende samtale forsøger at besvare. :9.

13.Jones SR, Carley S, Harrison M. An introduction to power and sample size estimation. Emerg Med J EMJ. september 2003;20(5):453–8.

14.San-José A, Pérez-Bocanegra C, Agustí A, Laorden H, Gost J, Vidal X, m.fl. Integrated health intervention on polypharmacy and inappropriate prescribing in elderly people with multimorbidity: Results at the end of the interven-tion and at 6 months after the intervention. Med Clínica Engl Ed. 26. marts 2021;156(6):263–9.

15.Coronado-Vázquez V, Gómez-Salgado J, Cerezo-Espinosa de Los Monteros J, Ayuso-Murillo D, Ruiz-Frutos C. Shared Decision-Making in Chronic Patients with Polypharmacy: An Interventional Study for Assessing Medication Appropri-ateness. J Clin Med. 24. juni 2019;8(6):E904.

16.Gregersen M, Jordansen MM, Gerritsen DL. Overall Quality of Life (OQoL) questionnaire in frail elderly: a study of reproducibility and responsiveness of the Depression List (DL). Arch Gerontol Geriatr. februar 2015;60(1):22–7.

17.Gerritsen DL, Steverink N, Ooms ME, de Vet HCW, Ribbe MW. Measurement of overall quality of life in nursing homes through self-report: the role of cognitive impairment. Qual Life Res Int J Qual Life Asp Treat Care Rehabil. au-gust 2007;16(6):1029–37.

18.Kalichman SC. Assessing medication adherence self-efficacy among low-literacy patients: development of a picto-graphic visual analogue scale. Health Educ Res. 14. juli 2004;20(1):24–35.

19.Wewers ME, Lowe NK. A critical review of visual analogue scales in the measurement of clinical phenomena. Res Nurs Health. august 1990;13(4):227–36.
