# Supplementary material for: Study protocol: The effect of a Medication Coordinator on the quality of patients’ medication treatment (MEDCOOR)—Randomized controlled trial
Source: PLoS One. 2024 Nov 26;19(11):e0314023. doi: 10.1371/journal.pone.0314023 (PMC11593754; doi:10.1371/journal.pone.0314023)
Supplement: S4 File — (PDF) [file pone.0314023.s004.pdf]

The effect of a medication coordinator on the quality of medication management: A randomized controlled trial

PhD student:

Maja Schlünsen, Cand. Pharm.

Principal supervisor:

Lene Juel Kjeldsen, Associate Professor, Research Leader, Ph.D.

Hospital Pharmacy Research Unit, Department of Regional Health Research,

Southern Danish University

Hospital pharmacy, Sønderjylland Hospital

Co-supervisor:

Trine Graabæk

Postdoc, Ph.D.

Hospital pharmacy Fyn, Odense University Hospital

Department of Health Services Research, University of Southern Denmark

Purpose

The purpose of the project is to investigate both the feasibility and effect of a medicine coordinator for hospitalized patients, measured by the number of potentially inappropriate medicines at discharge and 6 months after. The medicine coordinator starts from the patient's individual drug treatment and needs, with a focus on the transition from hospital to their own home. The medication coordinator's role is to act as the patient's and/or relatives' partner in connection with the optimization and dissemination of drug treatment. For this purpose, the booklet My medicine plan is being developed.

Research plan

The PhD project consists of three studies: 1. Development of the booklet My medication plan, 2. Evaluation of the feasibility of the intervention and 3. Randomized, controlled study (RCT) of medication review with medication coordinator.

Study 1 - Development of the booklet My medicine plan

In the project, it is desired to develop the booklet My medicine plan with space for thoughts, medicines, dosages, symptoms, future appointments, who is the doctor responsible for treatment, who should the patient contact, etc. (1,2).

Hahn-Goldberg et al have developed a discharge tool in which patients and relatives can note changes, discontinuation of medicines as well as future appointments in relation to consultations and what role the pharmacy may have(3). However, this tool is not adapted to a Danish context. In Denmark, the Patient's book was developed in 2006 – a guide to a safe patient procedure, which is a book of 131 pages with a wide range of good advice and space for notes from the patient and relatives(4). In this study, however, it is desired to develop a smaller booklet which could be a clear tool for the patient. For the development, semi-structured informal patient interviews(5) are carried out regarding the patients' discharge process in order to clarify the patients' expectations and wishes for information about medicines. Ten patients are expected to be included depending on data saturation. Furthermore, the role of doctors and nurses in connection with the discharge process is observed.

For the evaluation of My medicine plan, informal interviews are carried out with patients from the same department as the initial observations and informal interviews focus on whether My medicine plan can be helpful in the sector transition in order to obtain a relevant, usable and practical tool.

Study 2 – Evaluation of the feasibility of the intervention

Study 2 aims to evaluate the model for the medicine coordinator's intervention in connection with medicine reviews. It is the PhD student who is the medicine coordinator in the study. The medicine coordinator's role is as follows: To increase the patients' empowerment in relation to the use of medicines and thereby increase compliance, to act as a collaborative partner for the patient through mutual trust between patient and medicine coordinator, to carry out and communicate the results of the medicine review to the hospital doctor, who takes a position on the changes, and subsequently conveys any changes and the reasons for this

to the patient. In continuation of this, the medical coordinator must convey the epicrisis to the patient, so that the patient knows what information is passed on to the general practitioner(6–8). My medication plan, developed in study 1, is used in the process. It is therefore expected that the transition from hospital to home will be made easier, as the patient has aligned expectations in relation to which doctor is responsible for the course of treatment. Furthermore, in selected situations, the medicine coordinator must act as a link between the patient and general practitioner/specialist and thereby increase communication across sectors. The evaluation is carried out by assessing the process goals that occurred during the medication review. This is done by the medicine coordinator documenting agreed activities and their implementation. Furthermore, patient interviews are conducted with a selection of the included patients to obtain the patients' perspective on medication coordinator intervention.

2.1 Measuring points for the feasibility of the medicine coordinator intervention.

- Process goals:
    - How did we deliver the medication review?
    - Were there general needs that cut across the patients?
    - Were there individual needs?
    - Division of topics: compliance, concerns, inhalation technique etc.

• Which type(s) of inappropriate medicines were reduced based on the latest edition of Seponeringslisten(9) and Screening tool of older people's prescriptions (STOPP criteria)(10)

- Which medicines did the patients want to talk about during the medication review and which medicines did the patient actually want to stop taking.
- Which medicines were possibly never discussed?
- Satisfaction and implementation:
- What does the patient think of the contribution of a medicine coordinator?
- How can implementation of a medication coordinator be improved?

Study 3 – The effect of medication coordinator in connection with medication review

The purpose of this study is to investigate the effect of a medication coordinator, which is conducted as a randomized controlled trial, testing the following null hypothesis: There is no reduction in the number of listed PIMs between the intervention and control groups after medication review using the latest version of the Discontinuation List (9) and the STOPP criteria (10) after 6 months. The study will be conducted in accordance with CONSORT guidelines to ensure transparency and quality in the implementation and results of the project(11). The intervention is a complex intervention, where the various elements together make up the overall intervention. The intervention group receives a medication review in collaboration with a medication coordinator, while the control group receives standard treatment.

The patients will be included during hospitalization at Sønderjylland Hospital (SHS) by the PhD student in collaboration with the geriatric department. The course in the intervention group is illustrated in Figure 1.

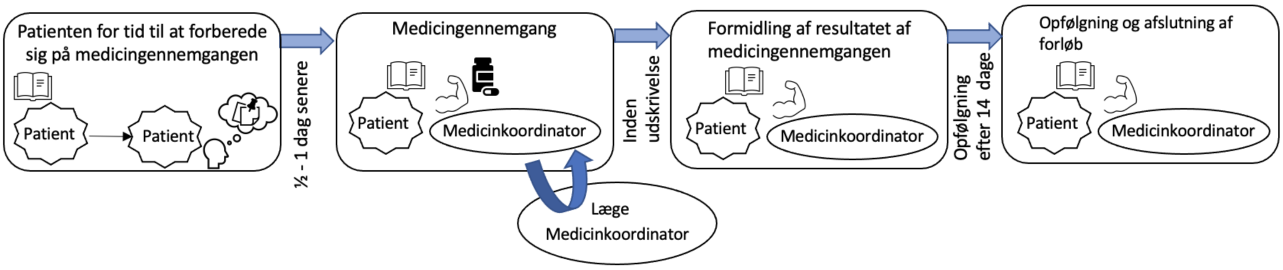

Figure 1. The course in connection with the medication review. The first box illustrates that the patients are given the booklet, My medicine plan, so that they have time to prepare for the medicine review. The second box illustrates the execution of the medication review and the medication coordinator's discussion with the hospital doctor. The third box illustrates the communication of the results of the medication review, and the fourth and last box illustrates the final follow-up between the patient and the medication coordinator. Symbols: the booklet illustrates My medication plan which is given to the patient, the arm illustrates patient empowerment. The medicine bottle illustrates the focus on drug treatment.

The patient is introduced to the medication review, which takes place ½ - 1 day later. This gives the patient the opportunity to prepare, as the patient is given material with questions, which can give rise to reflection in relation to the medication review.

The medication review is carried out using the motivational interview, where the focus is on the patient's wishes and the purpose of the drug treatment(12). In addition, the patient is encouraged to write notes in the booklet My medication plan. This is a way of making visible to the patient what the patient himself has expressed as wishes(12). The hospital doctor is informed of the changes/changes that the patient found important during the medication review and before discharge the result of the medication review is conveyed to the patient with the request to write down medication changes in My medication plan and to bring this with me at future consultations with the general practitioner. The medication coordinator contacts the patient around 14 days after discharge to follow up on agreements made in connection with the medication review.

The inclusion criteria are that the patient handles the dispensing and administration of medicines themselves and either administers 8 medicinal substances or 5 medicinal substances with at least 10 daily administrations.

3.1 The primary effect target for the intervention

The primary effect goal is to reduce the number of PIMs 6 months after discharge, assessed based on the drugs indicated on the latest version of the Discontinuation List(9) and the STOPP criteria(10). After 6 months, the patient's joint medication card (FMK) is assessed to see if changes made during the medication review are still valid, and whether discontinued treatment has been reintroduced.

The information that is obtained is the patient's current medicines at ATC code level. In addition, information is obtained about the patient's age and gender.

3.1.2 Strength calculation

Based on the literature, a power calculation is performed to assess the number of included patients based on the primary end point, which is PIMs assessed based on the Discontinuation List(9) and the STOPP criteria(10). The statistical program STATA is used for the calculation, where the power is set to 80% and a significance level of 5%(13). In the study by San-José et al, where the intervention was to discontinue PIMs and evaluate whether these remained discontinued after 6 months(14), a mean value for the number of PIMs

before the intervention was 1.4 with a spread of 1, 4 and a mean value of 0.7 PIMs with a spread of 1.1 after 6 months(14). According to the power calculation, this means that 52 patients must be included in each group. In the study by Coronado-Vazquez et al, an intervention is performed where the standard service is a medication review and the intervention is a medication review using patient involvement (15). The intervention group started with 1.39 PIMs and after the medication review this is reduced to 0.69 PIMs(15), which is why, according to the result of a power calculation based on this, 41 patients must be included in each arm. 67 patients must be included in each arm, if the starting point is the control group, where the starting point is 1.65 PIMs, which is reduced to 1.16 PIMs. In order to offset any dropouts, it is estimated that an extra 10% must be included, which is why, based on the most conservative strength calculation of 67 patients, it is expected to include 80 patients in each arm.

3.2 The secondary effect measure for the intervention

Furthermore, the patient's quality of life and perceived medication burden are examined. For this purpose, the questionnaire-measured Depression List(16,17) is used. This questionnaire contains 15 questions, which originally examine patients for possible depression, but the questions all relate to other aspects that have an impact on the quality of life(16). A visual analogue scale (VAS) is added regarding perceived medication burden(18). VAS can be used to assess subjective phenomena and these can thereby be converted to numerical data(19).

Ethical considerations

The Scientific Ethics Committee for the Region of Southern Denmark has been consulted in relation to whether the described project should be approved by the Scientific Ethics Committee. However, the three studies must be registered under the Region's Internal Register, as they work with sensitive personal data(8). Prior to participation in part-study 1, part-study 2 and part-study 3, the participants sign informed consent, which states that participation in the study is voluntary and which information is collected in each of the studies. It is made clear to the patients that they can withdraw their consent at any given time without giving an explanation,

Information and information obtained about the patients:

In sub-study 1, information is collected on patients' age, gender and the number of medicines prescribed in FMK. Furthermore, the patients are observed in connection with their discharge to gain knowledge about discharge interviews and an informal patient interview afterwards. These conversations are not audio-recorded, as observations are solely intended to gain an understanding of how discharges proceed and what is discussed in relation to the medication. Data is stored in anonymized form on a secured drive.

In part study 2, information is obtained about the patient's age and gender as well as their personal perspectives on the medicine coordinator service. These interviews will be audio recorded as verbatim quotes are needed. Data is stored in anonymized form on a secured drive.

In part study 3, information on the patient's age, gender and current medication list at admission, at discharge and 6 months after discharge at ATC code level is obtained. Additionally, patients answer a questionnaire regarding quality of life and perceived medication burden, where data is used to assess the effect of the intervention as a whole and not at an individual level. Data is stored in anonymized form on a secured drive.

References

1. Skovgaard AK, Fuglsang C, Graae EM, Haugaard IB, Christoffersen LB, Ljungmann R, et al. The health care system according to the Danes [Internet]. 2016 [referenced 23 January 2022] p. 72. Available at: https://www.tryghed.dk/viden/publikationer/sundhed/sundhedsvaesenet-ifoelge-danskerne

2.Danish Society for Patient Safety, TrygFonden. The patient's book - A guide to safe patient care [Internet]. 2nd ed. Vol. 2. Danish Society for Patient Safety and TrygFonden and Lindhard og Ringhof Forlag A/S; [cited 23 January 2022]. 131 pp. Available at: https://patientsikkerd.dk/content/uploads/2015/12/patientens_2.udg1opl.pdf

3. Hahn-Goldberg S, Chaput A, Rosenberg-Yunger Z, Lunsky Y, Okrainec K, Guilcher S, et al. Tool development to improve medication information transfer to patients during transitions of care: A participatory action research and design thinking methodology approach. Res Soc Adm Pharm RSAP. Jan 2022;18(1):2170–7.

4.Danish Society for Patient Safety, TrygFonden. The patient's book - A guide to safe patient care [Internet]. 2nd ed. Vol. 2. Danish Society for Patient Safety and TrygFonden and Lindhard og Ringhof Forlag A/S; [cited 23 January 2022]. 131 pp. Available at: https://patientsikkerd.dk/content/uploads/2015/12/patientens_2.udg1opl.pdf

5.Brinkmann S, Tanggaard L. Qualitative methods: a primer. Kbh.: Hans Reitzel; 2015.

6.Patient transitions - The Norwegian Agency for Patient Safety [Internet]. [cited 18 January 2022]. Available at: https://stps.dk/da/laering/risikoomraader/patientovergange/

7. Ministry of Health and the Elderly. Guide to Epicrisis [Internet]. Nov 30, 2018. Available at: https://www.retsinformation.dk/eli/retsinfo/2018/10036

8. Ministry of Health and the Elderly. Proclamation of the Act on scientific ethical treatment of health science research projects and health data science research projects [Internet]. Sep 1,2020. Available at: https://www.retsinformation.dk/eli/lta/2020/1338

9.Discontinuation list 2022 - recommendations for discontinuation of frequently used medicines in adults||Introduction [Internet]. [cited 18 January 2022]. Available at: https://app.magicapp.org/#/guideline/5773

10. O'Mahony D, O'Sullivan D, Byrne S, O'Connor MN, Ryan C, Gallagher P. STOP/START criteria for potentially inappropriate prescribing in older people: version 2. Age Ageing. 2015 Mar;44(2):213–8.

11. Cuschieri S. The CONSORT statement. Saudi J Anaesth. April 2019;13(Suppl 1):S27–30.

12.Rosdahl G. How can you motivate people to change behavior through conversation? This is the question that motivational interviewing tries to answer. :9.

13. Jones SR, Carley S, Harrison M. An introduction to power and sample size estimation. Emerg Med J EMJ. 2003 Sep;20(5):453–8.

14. San-José A, Pérez-Bocanegra C, Agustí A, Laorden H, Gost J, Vidal X, et al. Integrated health intervention on polypharmacy and inappropriate prescribing in elderly people with multimorbidity: Results at the end of the intervention and at 6 months after the intervention. With Clínica Engl Ed. 2021 Mar 26;156(6):263–9.

15.Coronado-Vázquez V, Gómez-Salgado J, Cerezo-Espinosa de Los Monteros J, Ayuso-Murillo D, Ruiz-Frutos C. Shared Decision-Making in Chronic Patients with Polypharmacy: An Interventional Study for Assessing Medication Appropriateness. J Clin Med. 2019 Jun 24;8(6):E904.

16. Gregersen M, Jordansen MM, Gerritsen DL. Overall Quality of Life (OQoL) questionnaire in frail elderly: a study of reproducibility and responsiveness of the Depression List (DL). Arch Gerontol Geriatr. 2015 Feb;60(1):22–7.

17. Gerritsen DL, Steverink N, Ooms ME, de Vet HCW, Ribbe MW. Measurement of overall quality of life in nursing homes through self-report: the role of cognitive impairment. Qual Life Res Int J Qual Life Asp Treat Care Rehabil. 2007 Aug;16(6):1029–37.

18. Kalichman SC. Assessing medication adherence self-efficacy among low-literacy patients: development of a picto-graphic visual analogue scale. Health Educ Res. 2004 Jul 14;20(1):24–35.

19. Wewers ME, Lowe NK. A critical review of visual analogue scales in the measurement of clinical phenomena. Res Nurs Health. 1990 Aug;13(4):227–36.
