# Supplementary material for: Study protocol: The effect of a Medication Coordinator on the quality of patients’ medication treatment (MEDCOOR)—Randomized controlled trial
Source: PLoS One. 2024 Nov 26;19(11):e0314023. doi: 10.1371/journal.pone.0314023 (PMC11593754; doi:10.1371/journal.pone.0314023)
Supplement: Attachment — Submitted filename: Respone to Reviewers.docx [file pone.0314023.s005.docx]

'Response to Reviewers'

Dear Editor and Reviewers,

Thank you for reviewing our manuscript and for your valuable suggestions and comments. Please find below our responses.

**Reviewer #1:** The authors designed a Randomized Controlled Trial to measure the Effect of a Medication Coordinator on the Quality of Patients’ Medication Treatment (MEDCOOR). However, the structure of the protocol does not fit with the guideline of the journal.

- First, thank you for all the comments and suggested changes. We have now revised the manuscript so it fits within the guidelines of PLOSONE.

Several breaks between paragraphs and unnecessary underlines hinder the quality of the paper.

- We have revised the breaks between the paragraphs and removed unnecessary underlines.

The authors did not define or operationalize “Quality of Patients’ Medication Treatment”. Therefore, the topic and the objective that is going to be measured are not parallel. I would suggest a careful review of the protocol and its feasibility before implementation. Publication of the protocol in its current form is barely considered.

- Thank you for your feedback. We recognize that a clear definition was lacking. Hence, we have clarified the quality of patients’ medication treatment throughout the manuscript by adding a definition and how this is related to PIMs for this study protocol. See line 71-83 in the introduction section and line 191-202 in the method section.

You might consider the following points to improve your work.
No Please reduce the number of keywords.

- Currently when re-submitting the manuscript, it is unfortunately not an option technically to reduce the number of keywords.
- We have removed: Polypharmacy, Patient care management, Empowerment, Quality of life, and Treatment burden.

Lines 33-34“communication of communicating suggested medication amendments to the patients” should be revised.

- Thank you for the suggestion. We have implemented this in line 42-44.

Line 190-192, Please describe the tool.

- We have included a brief description of the tool in the introduction section and 2.2.4.

Line 203-205, no tool validation information is included.

- The tool, *My Medication Plan,* has been developed for this study, where face validation was a part of the process. It has not yet been validated as such. This study is a part of the validation and further validations and improvements of the tool, *My Medication Plan*, are planned in the future.

**Reviewer #2:** Dear Authors
Thank you for your submission.
The comments will be available through journal editor.
Regards

- Thank you for your comments; they have been addressed throughout the revised manuscript based on the editor’s comment, which are found below.

**Reviewer #3:** The authors describe a protocol of a study which aims to evaluate the effect of a coordinating intervention on the quality of medication treatment. The primary objective of the planned study is the proportion of potentially inappropriate medication (PIM) six months after hospital discharge.

The description is detailed in many places; other points required by the SPIRIT guideline should be further elaborated.

- Thanks for this valuable input. We have adjusted the manuscript according to The SPIRIT Checklist where appropriate.
- A new Supporting Information File - SPIRIT has been uploaded.

Item 14; The sample size calculation in the manuscript is based on different assumptions and lead to a different number of patients to be includes (e.g. 93% power in the manuscript vs. 80% power in the study protocol resulting in n=70 patients per group in the manuscript or N=80 in the study protocol). Please explain deviations between the documents.

- Thank you for the comment. We apologize for the inconsistency, and we have adjusted the text according to advice by our statistician in the project group, hence the inclusion of 70 patients in the study protocol, but 80 in the original draft.

The sample size calculation based on the assumption that the expected proportions of PIMS in the intervention group is higher as in the control group. Please explain why this assumption is reasonable.

- We suspect that the intervention will focus on PIMs, and therefore the number of PIMs in the intervention is higher, since our intervention is better at identifying them.
- Furthermore, we based our calculation on a study where this was the case.

Item 15; Please add to the description of the clinic's resources which strategies will be used for achieving adequate participant enrolment to reach target sample size.

- This study is designed not to use any of the clinic resources, as the medication coordinator is an extra person. The intervention is intended to ease the workload of the clinic and support the clinic in relation to medication treatment.
- A paragraph to describe this has been added at the end of section 2.1.1.

Item 16a of the SPIRIT checklist suggests keeping details of any planned restriction (eg, blocking) in a separate document to conceal this knowledge to those who enroll participants or assign interventions. So the information from line 273 ff “A simple block randomization method …” should be transferred to a statistical analysis plan.

- As MS the main author of this article will assign, screen and recruit the patients, the information from line 273 and onwards cannot be concealed.

Item 16b; Not knowing the block length alone cannot avoid predictability. Further measures to enable concealment should be considered and described in the protocol.

- We have added a section in the manuscript concerning blinding of the statistician in section 2.6.

Item 16c; Details of the Implementation of the allocation list should be provided. Who will generate the randomization list with REDCap, who will enrol patients and who will assign participants to interventions?

- The first author will conduct all the components of the study. Hence, MS will screen, recruit and assign participants to the intervention – or control group. The randomization is performed through REDCap, with blocks of varying sizes, which have been decided by the data manager.

Item 20a; In general, the primary hypothesis, the sample size calculation and the statistical analysis should follow the same statistical approach. That means, if the primary objective is the comparison of the proportion of potentially inappropriate medication (PIM) six months after hospital discharge between the two treatment groups, the sample size calculation as well as the primary analysis should be based on the comparison of PIMs at month six. If other timepoint are considered as co-primary endpoints or as secondary endpoints should be decided and described. In the first case, a suitable multiple testing strategy should be considered. This strategy should then also be presented in detail! The likelihood ratio test across all time point does not prove the primary hypothesis. In addition, the sample size calculation should also focus on the contrast of the described model which address the comparison of PIMs at month six.
Moreover, some of the reads may be not familiar to the beta-regression approach. I would suggest explaining the concept with sufficient information to allow the analysis to be reproduced.

- As a consequence of your comment, We have decided that treatment effect should be evaluated across all time points, hence we evaluated that multiple testing is not necessary.
- We have added the following reference concerning beta regression: Ferrari SLP, Cribari-Neto F (2004). “Beta Regression for Modelling Rates and Proportions.” Journal of Applied Statistics, 31(7), 799–815.

Item 20b; The authors stated that no additional analyses are planned (20b n.a.) could you please outline which data will be analyzed by t-, Wilcoxon-, Fisher-, Chi-square- or log-rank- (Kaplan-Meier estimates) test.

- Thanks for your comment. We have now emphasized in the manuscript that the baseline variables will be analyzed using the above mentioned statistical tests and no additional analysis as described in the protocol will be conducted.

Moreover, some minor issues are to be noted;

The name of the intervention programme is used inconsistently, Please indicate the correct name MEDCORR or My Medication plan and please adjust throughout the protocol.

- The name of the intervention is MEDCOOR.
- *My Medication Plan* is a patient tool used during the medication review; hence it is a part of the intervention.
- We are not able to identify any mention of the intervention as *My Medication Plan,* but please let us know if this is the case after the revision.

The sentence on line 102 “The enrolment, intervention and assessment” is incomplete, please complete

- This sentence have been re-written, please see line 115-116.

Figure 1 in line 126 should be labeled as Figure 2

- All figures, tables, and supporting information have been updated to ensure correct citation.

Editors Comments:

Journal requirements:

When submitting your revision, we need you to address these additional requirements.

1. Please ensure that your manuscript meets PLOS ONE's style requirements, including those for file naming. The PLOS ONE style templates can be found at 
<https://journals.plos.org/plosone/s/file?id=wjVg/PLOSOne_formatting_sample_main_body.pdf> and 
<https://journals.plos.org/plosone/s/file?id=ba62/PLOSOne_formatting_sample_title_authors_affiliations.pdf>.

- The layout of the manuscript have now been XX to meet PLOS ONEs style requirements.

2. We note that the grant information you provided in the ‘Funding Information’ and ‘Financial Disclosure’ sections do not match. When you resubmit, please ensure that you provide the correct grant numbers for the awards you received for your study in the ‘Funding Information’ section.

- “Funding information” and “Financial Disclosure” match after revisiting both.
- Unfortunately, some of the grants to have a grant number.

3. Thank you for stating the following financial disclosure: 
 [(1) The University of Southern Denmark, one year salory to MS on 425.000 DKK and a grant of 10.000 DKK to be used on running cost. 
<https://www.sdu.dk/en/forskning/phd/phd_skoler/phdskolensundhedsvidenskab>
(2) The Region of Southrn Denmark gave one year salory to MS on 436.000 DKK 
<https://regionsyddanmark.dk/fagfolk/forskning/region-syddanmarks-forskningspuljer/ph-d-puljen>
(3) The University Hospital of Southern Denmark gave one year salory for MS on 572.000 DKK and a grant on 13.000 DKK to be used in running costs.  
<https://sygehussonderjylland.dk/>].  
Please state what role the funders took in the study.  If the funders had no role, please state: ""The funders had no role in study design, data collection and analysis, decision to publish, or preparation of the manuscript.""

- The role of the funder have been stated at the end of the section describing the financial disclosure.
- The funders had no role in the study.

If this statement is not correct you must amend it as needed. 
Please include this amended Role of Funder statement in your cover letter; we will change the online submission form on your behalf.

- The Role of Funder statement have been included in the revised Cover Letter.

4. When completing the data availability statement of the submission form, you indicated that you will make your data available on acceptance. We strongly recommend all authors decide on a data sharing plan before acceptance, as the process can be lengthy and hold up publication timelines. Please note that, though access restrictions are acceptable now, your entire data will need to be made freely accessible if your manuscript is accepted for publication. This policy applies to all data except where public deposition would breach compliance with the protocol approved by your research ethics board. If you are unable to adhere to our open data policy, please kindly revise your statement to explain your reasoning and we will seek the editor's input on an exemption. Please be assured that, once you have provided your new statement, the assessment of your exemption will not hold up the peer review process.

- As for now the only data we have to share is the Do File used for the power calculation (S2 File), as this is the study protocol.
- According to our ethics regulation, data of individual patients must not be exposed and available data will be anonymized.
- Do files used for the analysis will be made available.

5. Ethics statement only appears at the end of the manuscript:
Your ethics statement should only appear in the Methods section of your manuscript. If your ethics statement is written in any section besides the Methods, please move it to the Methods section and delete it from any other section. Please ensure that your ethics statement is included in your manuscript, as the ethics statement entered into the online submission form will not be published alongside your manuscript.

- The Ethics statement have now been included at the end of the Method Section, 2.8.

6. Please include captions for your Supporting Information files at the end of your manuscript, and update any in-text citations to match accordingly. Please see our Supporting Information guidelines for more information: <http://journals.plos.org/plosone/s/supporting-information>.

- Captions for Supporting Information files have been included at the end of the manuscript.
- Any in-text citation have been updated.
