# Supplementary material for: Study protocol: The effect of a Medication Coordinator on the quality of patients’ medication treatment (MEDCOOR)—Randomized controlled trial
Source: PLoS One. 2024 Nov 26;19(11):e0314023. doi: 10.1371/journal.pone.0314023 (PMC11593754; doi:10.1371/journal.pone.0314023)
Supplement: Attachment — Submitted filename: Respone_To_Reviewers.docx [file pone.0314023.s006.docx]

'Response to Reviewers'

Dear Editor and Reviewers,

Thank you for your suggestions and comments. Please see our answers.

**Reviewer #2**: Dear Authors
Thank you for revising the manuscript.
I hope the best outcome from this study that will be helpful for healthcare.
Best wishes

- Thank you for reading the manuscript once again, we appreciate it.

**Reviewer #3:** Thank you for the revised manuscript and the explanations. For me, some points remain open;

The authors clarified that the main author of the manuscript will assign, screen and recruit the patients. This approach is associated with a high potential for bias. The more important it is to take appropriate measures to avoid influencing the allocation. In particular, appropriate concealment measures should be implemented. Please note that concealment should not be confused with blinding. Concealment describes the non-disclosure of the assignment until the assignment itself, while blinding means the non-disclosure of the assignment beyond the assignment. Blinding of the statistician is not suitable to ensure concealment.

- Thank you for your comment. We agree that it is a concern that blinding is not possible; hence, there is a risk of bias.
- The allocation is random and concealed as the allocation is performed after the inclusion of the patient. The first author cannot influence the randomization.
- This study represents the best option; as other blinding methods were not feasible due to the intervention in itself.

The primary outcome is (re-)defined as (p. 9, line 206-207) “... the proportion of PIMs after hospital discharge. The PIMs are defined by four different screening tools.” Furthermore, the authors stated “As a consequence of your comment, We have decided that treatment effect should be evaluated across all time points, hence we evaluated that multiple testing is not necessary.” Nevertheless, it is still to be read (p- 12, line 310-311) that “To avoid inflating the type I error rate in the z-test, we will adjust the z-test for multiple tests.” Please clarify, which timepoint are actually included (In figure 1 it is indicated that PIMs are evaluated at t1, t2 and t6 – are this baseline, 3 and 6 months?), how the primary outcome is precisely defined (as change from baseline to any timepoint after discharge or value at any timepoint after discharge), and if adjusting the significance level for multiple testing will be an issue or not. In this context, it is equally important to make clear how the four different screening tools for defining PIMs will be applied and combined.

- Thank you for your comment. The primary analysis is a global test hence no adjustment for multiple testing will be utilized in that analysis. Denote that the analysis where we adjust for multiple testing is not the primary analysis but a secondary one, which only will be conducted if the global test is statistically significant. We have added on line 307 that the measurement is taken on baseline, at hospital discharge and 6 month after hospital discharge.
- The definition of PIMs is the amount of potentially inappropriate medication of the total medications of the given patient. A potential inappropriate medication is defined if at least one of the four tools indicates it. We have added/revised? the following formulation on line 207: “, hence if at least one tool indicates that a medication as a PIM, we will define the medication as a PIM”

In response to my question as to what the various tests mentioned are used for, the authors stated “We have now emphasized in the manuscript that the baseline variables will be analyzed using the above mentioned statistical tests and no additional analysis as described in the protocol will be conducted. “One of the recommendations of the CONSORT statement is that a table is presented showing baseline demographic and clinical characteristics for each group. Importantly, according to this recommendation in the CONSORT statement, “significance testing of baseline differences in randomized controlled trials (RCTs) should not be performed, because it is superfluous and can mislead investigators and their readers” (c.f. de Boer et al. International Journal of Behavioral Nutrition and Physical Activity (2015) 12:4).

- Thank you for you reflection. We agree that the usage of p-values in descriptive tables is inappropriate however in order to not restrict ourselves from a given journal, we will include p-values unless we are told otherwise or the author guidelines for the given journal states it clearly.
